# A Real-World Approach on the Problem of Chart Recognition Using Classification, Detection and Perspective Correction

**DOI:** 10.3390/s20164370

**Published:** 2020-08-05

**Authors:** Tiago Araújo, Paulo Chagas, João Alves, Carlos Santos, Beatriz Sousa Santos, Bianchi Serique Meiguins

**Affiliations:** 1 Computer Science Graduate Program (PPGCC), Federal University of Pará (UFPA), 66075-110 Belém, Brazil; carlossantos@ufpa.br; 2 Institute of Electronics and Informatics Engineering of Aveiro (IEETA), Department of Electronics, Telecommunications e Informatics (DETI), University of Aveiro (UA), 3810-193 Aveiro, Portugal; jbga@ua.pt (J.A.); bss@ua.pt (B.S.S.); 3 Computer Science Graduate Program (PGCOMP), Federal University of Bahia (UFBA), 40210-630 Salvador, Brazil; paulo.chagas@ufba.br

**Keywords:** chart recognition, deep learning, visualization, classification, detection, perspective correction

## Abstract

Data charts are widely used in our daily lives, being present in regular media, such as newspapers, magazines, web pages, books, and many others. In general, a well-constructed data chart leads to an intuitive understanding of its underlying data. In the same way, when data charts have wrong design choices, a redesign of these representations might be needed. However, in most cases, these charts are shown as a static image, which means that the original data are not usually available. Therefore, automatic methods could be applied to extract the underlying data from the chart images to allow these changes. The task of recognizing charts and extracting data from them is complex, largely due to the variety of chart types and their visual characteristics. Other features in real-world images that can make this task difficult are photo distortions, noise, alignment, etc. Two computer vision techniques that can assist this task and have been little explored in this context are perspective detection and correction. These methods transform a distorted and noisy chart in a clear chart, with its type ready for data extraction or other uses. This paper proposes a classification, detection, and perspective correction process that is suitable for real-world usage, when considering the data used for training a state-of-the-art model for the extraction of a chart in real-world photography. The results showed that, with slight changes, chart recognition methods are now ready for real-world charts, when taking time and accuracy into consideration.

## 1. Introduction

Data charts are widely used in technical, scientific, and financial documents, being present in many other subjects of our daily lives, such as newspapers, magazines, web pages, and books. In general, a well-designed data chart leads to an intuitive understanding of its underlying data. In the same way, wrong design choices on chart generation can lead to misinterpretation or later preclude correct data analysis. For example, wrong chart choice and poor mapping of visual variables can reduce the chart quality due to lack of relevant items, such as labels, names of axes, or subtitles. A redesign of those visual representations might be needed to fix these misconceptions.

With the original chart data available, it is possible to perform the necessary changes for mitigating the problems that are presented earlier. However, in the majority of cases, these charts are displayed as static images, which means that the original data are not usually available. Like so, automatic methods could be applied to perform the chart analysis from these images, aiming to obtain the raw data.

**Figure 1:**
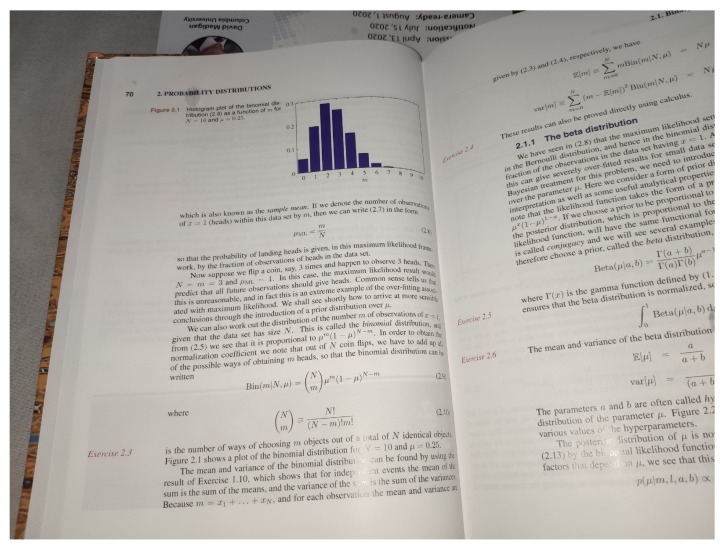
A Bar chart on real-world photography: tilted and in the middle of the text. The image is taken from a book [1]. Modern Chart Recognition methods do not cover real-world situations like this one.

When the input image contains other elements besides the chart image (text labels, for example), the detection of these charts must come as a prior step. This detection aims to locate and extract the data chart only, improving recognition performance. Additionally, in a real-world photograph, there is the factor of perspective to take into account. This factor means that the chart can be misaligned and might need some correction for the extraction step. Figure 1 illustrates an example of this situation. The chart is in the middle of a book page and slightly tilted, which indicates that it needs some perspective correction.

**Figure 2:**
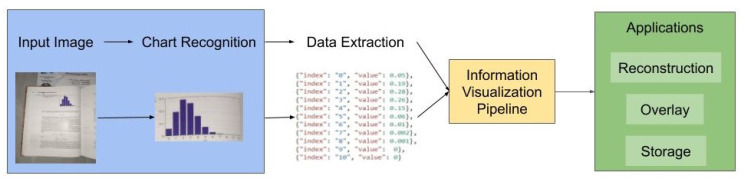
Automatic main process of chart recognition, from the input image to applications. The main process can transform a static environment in a rich user interface for manipulation of data. The information visualization pipeline is environment free, allowing the applications to be used in many environments. This is only possible when the initial step cleans the chart and gives information (blue area).

The process of automatic chart data extraction has two main steps [2,3]: chart recognition; and data extraction following a specific extraction algorithm for each type of data chart. This reverse-engineering for chart data extraction from static images is explored in the literature and softwares [2,4,5,6,7], with algorithms and methods for many chart types. After the data extraction, it is simple to rebuild the chart, while using a visualization library or software. The main process of automatic data chart extraction in Figure 2 covers the steps from the input image to the interaction with the rebuilt chart. The information visualization pipeline is the key of the main process in the reconstruction of new charts, as it can be directly applied on new environments, being used to overlay a new chart in place of the recognized one or simply store the data. This figure also highlights the fundamental initial step of chart recognition (blue area of Figure 2), as the focus from here onward.

Research papers regarding chart recognition usually focus on the type classification approach, while assuming a clean image to be classified as a specific chart type. Real-world situations are not that simple, the scenario where a user has a smartphone and wants to manipulate data from the chart is possible, as we have advances in many areas of research that soon will allow for the technology to get in this stage. The features of these scenarios and other real-world usage are not well defined by any modern work of chart recognition.

In this way, chart recognition can be defined as a process that is composed of three computer vision tasks: classification, detection, and perspective correction. Following the literature, the main usage scenario of chart recognition is to discover the chart type [2,3,5,8,9,10,11], using it to choose a proper data extraction pipeline. Nevertheless, even without data extraction, there are scenarios that chart recognition can be applied to. Take a set of digital documents as context, like a set of medical papers [12]. In this scenario, it can be useful to create metadata by chart type to support searches.

**Figure 3:**
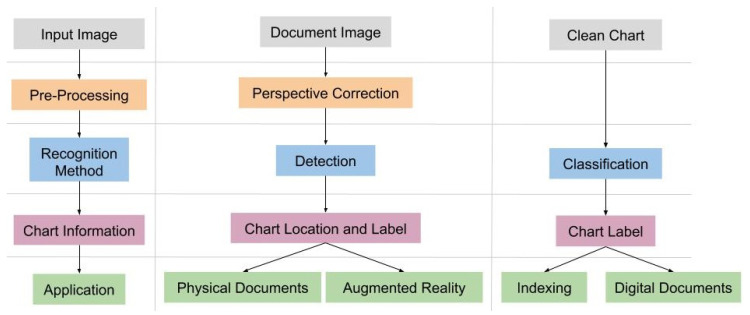
Process for Chart Recognition. Classification is common in literature, but other scenarios can be used if other vision tasks are aggregated. Chart Detection and perspective correction used together can make chart recognition more accurate and usable in new real-world scenarios, like Augmented Reality applications.

In the case where the documents have image tags (just as in some PDF and docx files), it is possible to apply classification algorithms to tag these files. However, if the chart is available as a raster-based image only, detection methods should be used. Webpages represent a similar scenario, since an online document can have image tags or SVG-based information for classification. For the printed documents scenario, the detection and perspective correction are fundamental steps to identify and correct chart images. The diagram presented in Figure 3 presents various scenarios and how each step of the process of chart recognition can be used on them.

Each step of this process of chart recognition brings many difficulties, for example, chart classification is complex due to variations not only between the different types, but also between charts of the same kind, which may differ in data distribution, layout, or presence of noise. Noise removal can be a challenging task, as the environment of the task dictates what is noise. In the context of chart recognition, two scenarios of noise are possible: the noise comes from real-world photography, so lightning and angle could generate an undesired effect on the images, and digitally with resolution loss by image softwares and screens. The other one is noise free, as some charts are generated by visualization libraries or softwares, and are directly integrated in digital documents.

Detection of a chart adds the bounding box search to the classification task, as it needs to locate the charts on a document file, report, or scanned print. Some works have addressed the standard approach for perspective correction through image rectification while using vanishing points [13,14,15,16]. However, to the best of our knowledge, none applied to the scenario of chart or document images for chart recognition. Some visual elements may appear in more than one type of chart, hindering the generalization of the classes. For example, consider two recurrent elements of line charts: lines and points. Lines can be found in Area charts, Arc charts, or Parallel Coordinates; points are also present in other chart types, such as scatter plots and some line charts [17]. Additionally, the legends and labels can be mixed with the context of the document, making it hard to locate and correct the chart image.

In the context of chart recognition, we propose an approach for classification, detection, and image rectification on static chart images. This work presents an evaluation of chart classification, paired with experiments on each task of the chart recognition process, namely, detection of charts on document images, and perspective correction using image rectification. The experiments were conducted with state-of-the-art techniques, using datasets of chart images collected from the internet and adapted for each task, with evaluations for each method, in order to analyze the efficiency and efficacy of adding these two steps in the chart recognition process. The results of our experiment presented accuracy in accordance with the most recent challenges in its respective areas. When considering each task’s results and the process at hand, it is possible to create applications and models that address the current needs of research on this area, such as preparing chart images for data extraction, image tagging for search, and usage on real-time scenarios. Classification and detection experiments use deep learning models for each step since these methods present outstanding results in several Computer Vision tasks. Furthermore, deep learning methods have been widely used by various chart recognition works [3,5,9,18,19]. Reverse engineering for chart data extraction from static images is explored in the literature and softwares, but it is not the focus of this work. It will be possible to use our process with data extraction methods (use the chart type, position to choose the right algorithms or guide the user, or both), as our process focuses on the starting steps of chart recognition.

Our proposal also used two scenarios of noise removal in the form of perspective correction. Because the classification step is based on clean images with no correction to be done, the detection step uses real document images with clean charts, and the perspective correction step using the distorted chart and document dataset. These scenarios represent the digital documents (no correction) and real-world images (perspective correction).

Moreover, as an example, with the advances of Augmented Reality (AR) technologies [20], it would be possible to recognize charts on the fly by applying the approach proposed here. With a mobile device, users can perform real-time chart recognition on a document and interact with it without changing the context. The usage of AR goggles will allow us to present virtual information directly into the user’s field of view while walking on a shopping center, comparing prices, or simply reading a report, providing seamless interactions with charts that were formerly static. This scenario is one use case of edge computing, as some processing could be done on a grander scale on edge than in a traditional cloud service. At the same time, advances are being made on the interaction of AR systems and in nanosystems to allow for in-place processing of these chart recognition models [21].

Despite our work having extensive experiment description sections, the main contributions of our work are to present a whole process for chart recognition that uses many computer vision tasks to cover different tasks. Highlighting the main scenario as recognizing charts in the real world and to present a real-world use case of an example working with no modifications from the trained models.

The organization of this paper is as follows: a rundown on common approaches and terminology for the problem of chart recognition is in Section 2, followed by related works in Section 3. The description of methods is depicted in Section 4, presenting dataset preparation, training regime, and evaluation metrics. The results are in Section 5 and a discussion of chart recognition process based in the results is in Section 6. Final remarks and future works are in Section 7.

## 2. Chart Recognition

Some Computer Vision tasks are complex, demanding a high level of abstraction and speed, like classification, tracking, and object detection [22]. A natural way to deal with these problems is to use a technique that admits grid-like data as input and does not need a specific feature extractor [23], learning representations with a dataset. Convolutional Neural Network (CNN) is specialized in these requirements and it has achieved excellent results on image classification and other tasks [24].

### 2.1. Image Classification

In the context of chart image recognition, there has been a focus on the task of image classification, which consists of categorizing a static input image based on a chart image dataset of chart images. Throughout the years, computer vision methods followed the classical methods until the advent of deep learning [2,3].

The image classification task shows the efficiency of representation learning through deep learning as compared to the classical approaches. Classical methods for image classification use handcrafted feature extractors paired with a machine learning classifier. This way, even when the feature extractor is robust, intrinsic spatial data are lost if not explicitly extracted. Furthermore, feature extractors are not universal, so each computer vision problem required manual engineering of features [23]. Thus, CNNs are the current state of the art for image classification tasks.

A CNN groups the filters into hierarchical layers, learning complex and abstract representations from the dataset [25], and orders the filters as layers. State-of-the-art architectures are being used on recent works for chart recognition [3,5,9,19], focusing on the ones that won the ILSVRC challenge [24]. The main ones, which are present in most deep learning textbooks and courses, are: VGG [26], ResNet [27], MobileNet [28] and Inception [29].

The evaluation of these architectures usually follows traditional classification accuracy metric, while using the inference results and comparing with the ground truth labels. In more detail, top 1 accuracy uses the best class of the inference and compares with the ground truth, and the top 5 accuracy uses the range of the best five classes to compare with the target label.

### 2.2. Object Detection

Object detection is one of the most challenging problems on computer vision, comprehending both classification and localization of objects in an image [30]. In this task, the classification is extended with the bounding boxes’ regression, as the identified objects must match in their respectively ground-truth position. A straightforward solution for this task is sliding windows of predefined sizes in all areas of the image and classify each patch. However, this is computationally expensive, making impossible its use on real-world applications. CNN-based solutions can tackle this issue, extending grid-like data processing to object location.

Detection frameworks that are grounded in CNN methods are presenting outstanding results on various object detection domains [24,31,32], as they can learn localization information along with object feature information. These frameworks have a neural network backbone that works as a CNN feature extractor for classification. This backbone can be any CNN model (e.g., Inception, ResNet, or VGG), and its computation can be shared depending on the implementation. There are two main types of these frameworks: one stage detectors and two stage detectors. One stage detectors are fast enough to use in some real-time scenarios, as it speeds up computation without losing accuracy, like RetinaNet [33]. Two-stage detectors usually provide stable results, but they have slower inference time when compared to one-stage detectors, Faster R-CNN [34] being one of them.

The evaluation of object detection frameworks is usually done while using the same metrics of the MS-COCO Recognition Challenge [31], which are the Average Precision (AP) and the Average Recall (AR), both with different scales and thresholds. The AP metric is a relation between precision and recall, and it is separately computed for each class and then averaged. The metric calculation, which uses the true positives and the false positives (for precision and recall), consider two criteria: the predicted class; and the Intersection over Union (IoU) ratio, which measures the overlap of the predicted bounding box and the ground truth bounding box in a certain threshold. If a certain object has its class predicted correctly, and the bounding box predicted IoU is over the threshold, then it is a true positive; otherwise, it is a false positive. The AP is also known as mean Average Precision (mAP), and, in this work, they are equivalent. The equations for AP and IoU are exposed on (1) and (2), respectively, with the variables: $C_{r}$ the total of classes, *c* a class, $P_{b}$ the predicted bounding box, and $T_{b}$ is the ground truth bounding box.

(1) $$AP=\frac{1}{Cr}\sum_{Cr}\sum_{c}precision(c)\times \Delta recall(c)$$

(2) $$IoU=\frac{Area(P_{b}\cap T_{b})}{Area(P_{b}\cup T_{b})}$$

The COCO challenge defines different average precision notations for different IoU thresholds, notably for an average of 10 values of IoU threshold values ranging from 0.5 to 0.95 with a step of 0.05 (notated as [0.5:0.05:0.95] from here onward); the 0.50 threshold; and, the 0.75 threshold. These metrics are notated, respectively, as AP (for IoU=[0.5:0.05:0.95]), $AP^{IoU=.5}$ and $AP^{IoU=.75}$.

### 2.3. Perspective Correction

The perspective correction has been applied in many computer vision tasks, such as automobile license plate recognition, non-Latin characters OCR, and document rectification [35]. These corrections are applied to perspective distortions that can be found in real-world photography, as digital cameras follow a pinhole camera model that generates it [36]. Real-world chart recognition is subject to these perspective distortions and also of its corrections.

Techniques for perspective correction have been widely used in real-world situations in the scenario of planar document rectification, where a distorted document is corrected for future processing, mostly OCR. Chart detection models frameworks could benefit from a rectified document image before performing object detection, as the image comes from a digital camera. For example, it is possible to use some approaches directly of image rectification over photos to chart document rectification.

Image rectification is the reprojection of image planes onto a common plane, and this common plane is parallel to the line between camera centers. Formally, given two images, image rectification determines a transformation of each image plane, such that pairs of conjugate epipolar lines become collinear and parallel to one of the image axes [13]. A way of achieving this is through homography transformations.

The homography is a transformation that defines a relationship of two images on the same plane. This transformation can be used to rectify an image, given relationship hints of the distorted image with its rectified version. One way of achieving this is discovering the vanishing points of an image and using these points to estimate homography between the distorted image and its rectified version.

While vanishing points for homography estimation is present in many methods of image rectification, the method for finding these vanishing points can vary from simple methods to more robust ones. An example of simple methods is matching epipolar lines directly [13] or finding parallel lines [35]. Additionally, robust method examples are searching edgelets [14], using RANSAC on radon transformed images [15] or even training a neural network [37].

Usually, the evaluation is done by comparing images or counting correctly recognized words by OCR software [37,38,39]. In cases where one has the homography matrices that distorted the images, an evaluation of errors can be measured with an error metric, like Mean Absolute Error (MAE), since it can be used to measure an estimator.

## 3. Related Works

Several works have been developed on the topic of data chart image classification and detection. These tasks have gained attention, mainly due to its importance in the automatic chart analysis process. Following the traditional image classification pipeline, Savva et al. [2] presents Revision, a system that classifies and extracts data to recreate charts. The dataset used has 2500 images and is collected from the internet and it is composed of 10 classes—area charts, bar charts, curve plots, maps, Pareto charts, pie charts, radar plots, scatter plots, tables, and Venn diagrams. A set of low-level image features and text-level image features were used as input of an SVM classifier, with an average accuracy of 80%. Our work and many others follow this concept of collecting datasets from the internet.

Jung et al. [5] proposed the ChartSense, an interactive system for chart analysis, including the chart classification and data extraction steps. They also used CNNs for classification, comparing three well-known models from the literature: LeNet-1, AlexNet, and GoogLeNet. The models were evaluated while using the Revision dataset. For final classification, more images were collected and added to the Revision dataset, achieving the best accuracy of 91.3% while using GoogLeNet.

Chagas et al. [9] proposed an evaluation of more robust CNN models for chart image classification. Unlike the previously cited works, the proposed methodology has two main tasks: training using synthetically generated images only, comparing the CNN models with conventional classifiers, such as decision trees and support vector machines. The proposed approach aimed to evaluate how the models behave when training with "clean" generated images and testing on noisy internet images. They used a 10-class dataset (arc diagram, area chart, bar chart, line chart, parallel coordinate, pie chart, reorderable Matrix, scatter plot, sunburst, and treemaps) with 12,059 images for training (approximately 1.200 instances for class) and 2683 images from test, evaluating three state-of-art CNN models: VGG-19, Inception-V3, and Resnet-50. The best result was the accuracy of 77.76% while using Resnet-50.

The work of Dai et al. [3] uses few classes (Bar, Pie, Line Scatter, and Radar) than ChartSense, Revision, and the work of Chagas et al., but with accuracy around 99% for all CNNs evaluated. The dataset is also collected from the internet, it has 11,174 images with semi-balanced instances for classes, and the work follows the classification with data extraction. In this context, CNNs showed state-of-the-art results throughout the years for the problem of chart image classification, and our work extends the classes of charts used (10 for 13), followed by a straightforward parameter selection for the state-of-the-art architectures.

Although some works have addressed the chart analysis problem, most of them focused on the chart classification and data extraction tasks, while only a few approached the chart detection issue. Kavasidis et al. [10] introduced a method for automatic multi-class chart detection in document images using a deep-learning approach. Their approach used a trained CNN to detect salient regions of the following object classes: bar charts, line charts, pie charts, and tables. Furthermore, a custom loss function based on the saliency maps was implemented, and a fully-connected Conditional Random Field (CRF) was applied at the end to improve the final predictions. The proposed model was evaluated on the standard ICDAR 2013 dataset (tables only) [40], and on an extended version with new annotations of other chart types. Their best results achieved an average F1-measure of 93.35% and 97.8% on the extended and standard datasets, respectively.

Following a similar path to chart detection, some works have been tackling the table recognition task on document images. Gatos et al. [41] proposed a technique for table detection in document images, including horizontal and vertical line detection. Their approach is only based on image preprocessing and line detection, not requiring any training or heuristics. Schreiber et al. [42] developed the DeepDeSRT, a system for detecting and understanding tables in document images. Their work used Faster R-CNN architecture, which is a state-of-art CNN model for object detection. The proposed model was evaluated on the ICDAR 2013 table competition dataset [40] and a dataset containing documents from a major European aviation company. Document images are used in our work to build a chart detection dataset with chart overlay.

The primary goal of chart detection is finding the localization of the chart image on the input image, which is usually a document page. Huang and Tan [43] proposed a method for locating charts from scanned document pages. The strategy of their work is finding figure blocks from an input image and then to classify this figure in a chart or not. The figure localization used an analysis of logical layout and bounding box, and the image classification is based on statistical features from charts and non-chart elements. Even though their method does not return a specific chart type, the proposed approach achieved promising results, obtaining 90.5% of accuracy on figure location. For figure classification, the results were 91.7% and 94.9% of precision for chart and non-chart classification, respectively. Their work focuses on finding charts and does not fall on the direct definition of multi-class object detection used in our work.

Multi-class chart detection in document images is still an active field of research. One major challenge in this field is defining relevant features for classifying different chart classes, which may vary depending on specialist skills or chart types. This way, deep-learning methods have the advantage of not relying on hand-crafted features or domain-based approaches [23]. Accommodatingly, recent papers have used deep-learning-based architectures for chart classification; in this way, the work presented in this article uses more classes (13) and more images (approx. 21.000) as well as chart detection and perspective correction.

These papers cover specific steps of chart recognition, while allied with some other steps from the main process of chart recognition, extraction, and reconstruction. We take influence on many aspects of these works, like the dataset collection, the classes division, and the chart overlay on document approach, despite that, different from the previous works, our work covers all of the steps from the chart recognition, filling a gap of a complete process to compute static chart image into information. In addition, it also introduces a real-world example of chart recognition of charts on a book.

## 4. Methods

Most of the choices for the methods used in this work are based on the challenges that emerged from the following tasks, ImageNet for classification, MS-COCO for detection, and ICDAR dewarping for perspective correction. These are hard challenges that proved the efficacy of these models. The methods that are used to train the models, hyperparameter selection, dataset collection, and evaluation are described in the next subsections.

### 4.1. Datasets

A chart dataset must cover significant differences of each chart type. Data aggregation, background, annotations, and visual marks placement are visual components that vary from chart to chart, even as the same class. This variability is expected and some authors [2,3,5,18,44] address this variability on the collection step, searching the images from the internet, where chart designers publish their work in various different styles. While some datasets could be used for training and evaluation of these techniques, as the ReVision dataset [2] or the MASSVIS dataset [45], we choose to collect data from the internet to use a large number of images to train the methods.

The dataset collection step of this work follows the approach of [3], downloading the images from six web indexers: Google, Baidu, Yahoo, Bing, AOL, and Sogou. The chart types used are arc, area, bar, force-directed graph, line, scatterplot matrix, parallel coordinates, pie, reorderable matrix, scatterplot, sunburst, treemap, and wordcloud with the following keywords (and its chinese translations): arc chart, area chart, bar chart, bars chart, force-directed graph, line chart, scatterplot matrix, parallel coordinates, pie chart, donut chart, reorderable matrix, scatterplot, sunburst chart, treemap, wordcloud, and word cloud. More than 150,000 images were collected using these queries, and we kept only the visualization that falls on the following criteria: two-dimensional (2D) visualizations, not hand drawing, and no repetitions. The total of images downloaded that falls in our rules was 21,099, and the summary of the dataset is in Table 1, with its respective train/test split. The split process was automatically done by a script on the image files, and the training split ranges from 85% to 90%, depending on the number of instances of the class. All 13 classes are used for all of the experiments.

The selected types cover most usages of visualizations. The bar chart, line chart, scatterplot, pie chart, and word cloud are chosen, as they are broadly used [4]. Sunburst and treemap are hierarchical visualizations, reorderable matrix, and scatterplot matrix are a multi-facet visualization type. Area and parallel coordinates are multi-dimension visualizations, and arc and force-directed graphs are graph-based visualizations. The selection of these types covers most users’ needs. Some classes have few images, as they are not as popular.

The classification experiment uses the downloaded images, ratio scaled and padded to (100 × 100) size, randomly received augmentation on shear, and zoom by a factor of 0.2 and a 0.5 chance of horizontal flipping, and the pixel values are normalized to be in the –1 and 1 range. For the detection dataset, context insertion is used to create a scenario for chart detection close to a real document page. For this step, the generated charts are overlaid over real document images. Some works used similar approaches, showing results that were at par with the classic approaches [46,47,48].

The charts were uniformly located entirely in the document image. In some documents, scale transformation is used, by 1/2 or 1/4 of the size of charts. The size of the document images is scaled to 1068 × 800, where the charts have dimensions that vary from 32 × 32 to 267 × 200. The document images used in this work are from the Document Visual Question Answering challenge in the context of CVPR 2020 Workshop on Text and Documents in the Deep Learning Era [49], which features document images for high-level tasks.

**Figure 4:**
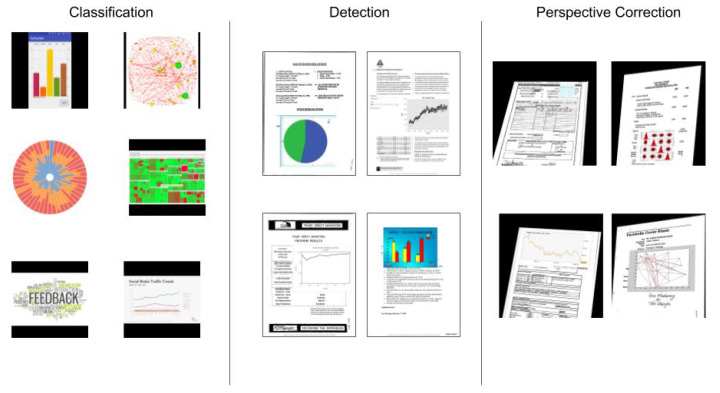
Samples of three datasets for the experiments (from left to right): classification, with added chart images; detection, with chart overlaying document images; and, perspective correction, with distorted images.

A distorted images test dataset of the detection experiment is used for the perspective correction experiment. These distortions are applied while using homography matrices generated with a simple method of perturbation, where a factor of 2 moves each corner of the document image and a homography is calculated with this new distorted image. Figure 4 shows samples of the three datasets.

### 4.2. Training and Evaluation

The most common training approach for deep-learning applications uses a pre-trained model and then retrains the model on a new domain dataset. This strategy also applies to CNN classification and detection problems, aiming to exploit features learned on a source domain, leading to a faster and better generalization on a target domain [50]. For this work, the models were pre-trained on the ImageNet dataset [24] for classification and MS COCO [31] for detection. This retraining step is also called fine-tuning, where some (or all) layers must be retrained, adapting the pre-trained model to the chart detection domain. We chose the transfer-learning approach based on fine-tuning the entire network on the target domain. For object detection, this could be done in two ways: with a pre-trained backbone only or with the whole network pre-trained, including the object boxes subnetworks. We chose the pre-trained backbone on ImageNet, because it allows results that reflect some common use cases.

The backbone can be fine-tuned from a large-scale image classification dataset, such as ImageNet. The features can be easily transferred to the new domain, since the backbone is necessarily a set of convolutional layers that can identify features, just like in the classification domain. The subnetworks for box prediction are fine-tuned similarly, but using the knowledge of the region proposal stage (for two-stage detectors) or using the last layers of the convolutional body (for one-stage detectors) to improve box location precision.

For both classification and detection experiments, no mid training changes were used (early stopping, schedule for learning rate changes). They followed the default parameters of the engines unless explicitly stated. The models were trained and evaluated in two different machines, classification and perspective correction in one computer with a GTX 1660 with 6 GB of memory, and the detection experiment ran on a computer with a Titan V video card with 12 GB memory. The engines used for the training (Tensorflow [51] and PyTorch [52]) allow for the training of the models in one machine and run on others with different configurations, given some engine restrictions. It is not decisive for the following sections after training.

#### 4.2.1. Classification

The classification experiment evaluated four different CNN architectures: Xception [53], VGG19 [26], ResNet152 [27], and Mobilenet [28]. These architectures have been chosen, as they are considered to be classic in the literature and they are available in most deep learning frameworks [51,52,54].

Their weights are pre-trained on the ImageNet dataset [24], and Hyperparameter selection is used, the training of the five models for architecture is done in a random search fashion, tunning learning rate, and weight decay with values $\left[10^{-4},10^{-5},10^{-6}\right]$ and $\left[10^{-6},10^{-7}\right]$, respectively, for 30 epochs in batches of 32 images each.

Classification evaluation is done by measuring accuracy on the test set, picking the best prediction of the CNN. The evaluation is done over all classes, and separately on four classes: bar, pie, line, and scatter. These chart types are popular, and they can be used as an estimate to comparison with other works [2,3,18]. All of the models are evaluated using top-1 accuracy.

Tensorflow 2 [51] is used as the Deep Learning engine for training and evaluation. Datasets are loaded and augmented while using native Tensorflow 2 generators. This experiment ran on a GTX 1660 6 GB video card on an 8 GB memory computer.

#### 4.2.2. Detection

The detection experiment evaluated two distinguished object detectors: RetinaNet [33] and Faster R-CNN [55]. The backbone CNNs are ResNets pre-trained on the ImageNet dataset, and the weights of the whole models were pre-trained on the COCO dataset [31], following the work of the original authors. We choose two one-stage detectors that present state-of-the-art results on COCO and Pascal VOC datasets [56], following our premise of using fast methods for detection inference, in order to enable real-time applications. Hyperparameters of the two detectors are used, as defined by the original authors, only changing the batch size to four images and the iterations for 90,000 (approximately 20 epochs).

The evaluation of these detectors is done while using the COCO challenge metrics alongside inference time. The inference time is a critical metric for object detection, since real-time applications can use fast detection in various tasks. It can be computed as the time in seconds that the framework process the input image and returns the class and the bounding box of the objects on the image. Hence, the frameworks process the input image returning the class and the bounding box of the objects on the image. For this work, the frameworks are evaluated while using the AP, $AP^{IoU=0.5}$, $AP^{IoU=0.75}$, and the inference time.

We used the original authors’ recommended engine for the implementation of the selected detectors. RetinaNet and Faster R-CNN frameworks are implemented in the Detectron 2 [57] platform, its implementation is publicly available, runs on the Python language, and it is powered by the PyTorch deep-learning framework. Detectron2 is maintained by the original authors of RetinaNet and Faster R-CNN. This experiment ran on a Titan V 12 GB video card on a 64 GB memory computer.

#### 4.2.3. Perspective Correction

The method for perspective correction follows an image rectification approach. Only one method is evaluated once the ready to use ones are not available, and they are not easy to implement from scratch. Also, commercial approaches have data sharing and usage restrictions. The chosen method is a slight variation of the work of [14], and it is available online in [58]. This method estimates the vanishing points to compute a homography matrix to rectify the original image.

The evaluation applied MAE to measure the estimated homography between the ground truth and the distorted image. The assessment considered three scenarios: raw homography, no scaling, and no translation. Some real-time scenarios could benefit from controlling the scaling and position at will without it being imbursed on the transformation. The experiment ran on a 32 GB memory Intel core i7 machine.

## 5. Results

We present the results of each individual step using recent state of the art of the art methods. The discussion is provided at the end of the section.

### 5.1. Classification

The classification step shows remarkable results in different conditions. The best models present results for accuracy over 95% results corresponding to all classes (13) and only four classes. The results for the four classes are overall slightly better than 13 classes, but it uses only chart types with a great number of samples. Table 2 shows the best two models of each architecture. The best model is an Xception with a learning rate of $10^{-4}$ and a decay of $10^{-6}$. The other architectures have an error margin of no more than 3.5% as compared to the best, showing that the moderns architectures could be used if some other task is needed to do so. This result indicates that finetuning the models with little hyperparametrization can deliver good results in this task.

The confusion matrix prsened in Table 3 shows the best Xception model performance for each class and the most common errors over the test set. The scatterplot matrix chart had the most errors than any chart class, with errors pointing to force-directed graph and scatterplot. This mismatch shows that some characteristics of the layout organization are being lost. Arc charts have no errors, and no other class missed itself for it. The mistake could be a clue of a distinct chart type with little data.

### 5.2. Detection

RetinaNet presented the best values for all APs, endorsing the use of Focal Loss for precision improvement on detection. Furthermore, being a one-stage detector also brought the best result for inference time. More training time could be necessary to achieve better results, as Faster R-CNN is a two-stage detector. The inference time for both methods is below 0.25 seconds per image. Given the high resolution of the images and the framework used alongside the video card, it is acceptable for some applications. Table 4 shows the overview results of the detection experiment.

The AP results for each class follow the total AP shown in Table 5, except for the arc chart and wordcloud classes. This discrepancy of the values for Faster R-CNN and RetinaNet does not comply with results from the literature on other challenges, where RetinaNet is faster, but Faster R-CNN has better AP [33] overall. Our work showed that RetinaNet got better results with time and AP. We did not make any hyperparametrization besides batch size and number of epochs and this might produce results that are more in line with the expected from the literature with cautious hyperparameter search. However, this is beyond the focus of this work. It is important to notice that this time is of the evaluation alone, and it is not from the next section results.

### 5.3. Perspective Correction

The rectification experiment for perspective correction presents three scenarios: the estimation of the raw homography (no changes on any parameter), homography without scale, and homography without translation. The MAE from the raw homography and homography without scale had a similar average, 33.16.

We highlight the results that were obtained with homography without translation, as the average value of 0.12 achieved by the method showed that document positioning on the new rectified plane generates more errors because removing the translation from the estimation removes most of the errors. It is essential to notice that the position is not decisive for this process, once it is only is a preprocessing step for chart detection, and it can be safely ignored in most cases.

### 5.4. Discussion

The process of chart recognition can be used in many scenarios, such as indexing, storage of data, and real time overlay of information. While many works [3,5,9] focused on chart classification, only a few addressed the chart detection problem on documents [10,11]. The chart detection in documents can use general approaches of other vision tasks for it context, as we used state-of-the-art models and methods of the MS-COCO challenge, and it can be amplified enough to use techniques of document analysis research field, like the approaches of real-world photography in document images. Even so, the first experiment is a chart classification, once some works did it with fewer classes than others [3], using different methods [9], and not presented parameter selection.

Various works have used a dataset collected on the internet, which is more important than the classification method chosen, as the classification step’s difference is minimal for each CNN architecture. For example, Chagas et al. [9] used synthetic datasets for training and internet collected for testing, with ten classes, and using the same architectures. The results showed that there was a difference in the accuracy of training and testing with the internet collected dataset is above 15%. In this context, some studies regarding hyperparameter selection must be performed, but it should not be exhaustive given a reasonable amount of data.

The results of the classification experiment showed that state-of-the-art architectures could perform very well given enough data, even for the problem of chart classification with many classes. The work of [3] already showed this with four classes, and we expanded it to 13 classes, while using more recent CNN architectures. The safety that is given by these methods allows for interchangeably using these architectures for other tasks aside chart classification, which usually uses a CNN classifier. For example, ResNets are backbones on many detection frameworks [57]. The ImageNet trained Inception architecture is used on the base example of DeepDream [59] application. The MobileNet architectures [28] are small and fast. The loss function of SRGAN is based on VGG19 feature maps [60]. One could choose the best architecture and train a chart classifier to bootstrap another task.

Despite that detection did not reflect the results of literature, it showed that with little to none hyperparametrization, it is possible to train a detector that acquires AP good enough in document scenarios. Although it lacks a dataset of real-world charts annotated, the training of a method using a chart overlay can be successfully used in real-world scenarios, as shown in the next subsection.

Perspective correction presented good results, with low MAE for the non-translation scenario. Some image rectification solutions are industry-ready, embedded in some applications [61], and using a perspective correction on the process of chart recognition looks a natural next step on the document analysis scenario. The implementation of this process also guarantees that old pipelines do not break, as data extraction methods require rectified images. Other image corrections could also be applied with no extra tooling.

One application of the process of chart recognition process proposed can be a real-time use of these methods, as stated in the introduction. While using a Titan V video card, it is possible to detect charts in almost real-time, so for most high-end specs cards, it is possible to use this process on these time-intensive applications [62]. Even if it is not acceptable for frame-by-frame real-time use in time-intensive applications [63], some shortcuts can be used, such as frame skipping, resolution scaling, and object tracking to minimize the perceived latency for the users. For an augmented reality mobile application, a high-end video card could be part of a cloud service that does the heavy computation, allowing for the mobile device to position the results correctly.

## 6. Use Case

**Figure 5:**
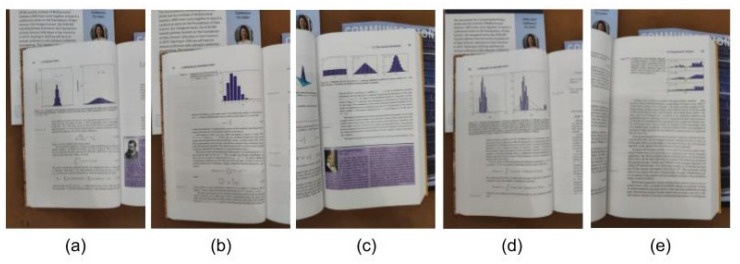
Bar chart photographs taken from a book [1] and transformed for evaluation. (**a**,**d**) present two bar charts with text, (**b**) shows one bar chart, and (**c**,**e**) present three bar charts. For this evaluation, the detector considers only the most accurate detection.

We propose a task of detecting real-world charts in documents using the models trained in our work, and of the best of our knowledge, there is not any annotated dataset for this task. We chose a simple evaluating metric: full detection and partial detection of a chart image. The first one detects all of the charts and no text outside of it, and the second one detects only part of the chart, or there is some text outside it. Only the highest score of the full detection is used. We choose the Bishop’s book [1] as our physical document and manually searched all of the bar charts with axes (most popular chart for several uses [4]), and took photographs of them. The images for this task are displayed in Figure 5.

These photographs are transformed by rotations from −4 to 4 degrees with step 0.5, while using the center as the pivot, summing 16 (original + 15 transformations) images for each book page. Two modes are evaluated: a normal mode, with no rectification, and one with rectification, with a total of 160 images at the end. The results are shown on Table 6.

Even with rectification, some charts are very hard to detect (Figure 5a,d), which implies that even using synthetic overlayed charts, more transformations should be applied. For example, the pure white pages used do not reflect the reality of white from photographs, that receive heavy light influence, as well as more images resolutions to capture the quality of high-end digital cameras. Even so, the rectification results showed that the image preprocessing leads to better results.

### Illustrative Example

**Figure 6:**
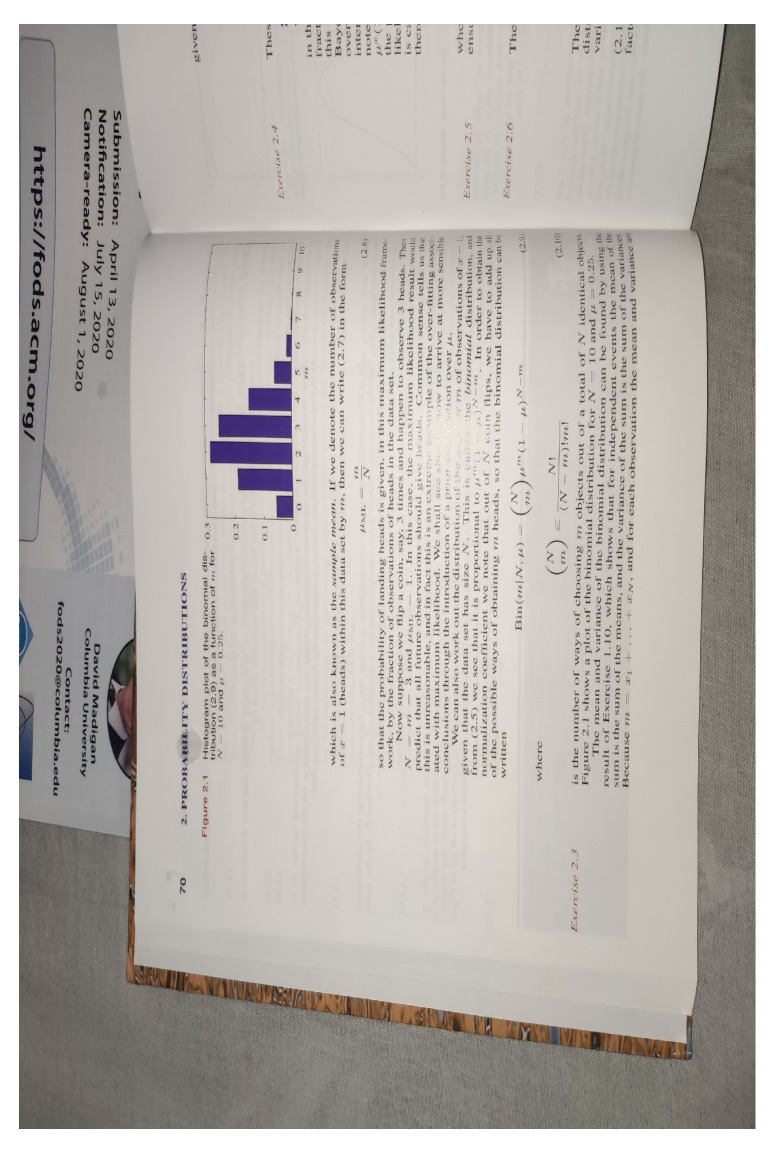
Input image from the use case. The bar chart must be located and extracted.

A single example of a user scenario can showcase the complete step by step chart recognition process. The goal of this example is, given a real-world photograph with a bar chart, to highlight the bar chart position, following the previous use case. This example computes the perspective correction of a real-world photograph with a chart image and detects the position. All steps of this process are executed in a single machine, with a GTX 1060 video card with 6GB of RAM. It is not a high-end video card, but it compensates for its cost. When considering the real world, it is safe to assume that the process will not always have access to the high-end video card specs all the time. The input image is shown in Figure 6.

**Figure 7:**
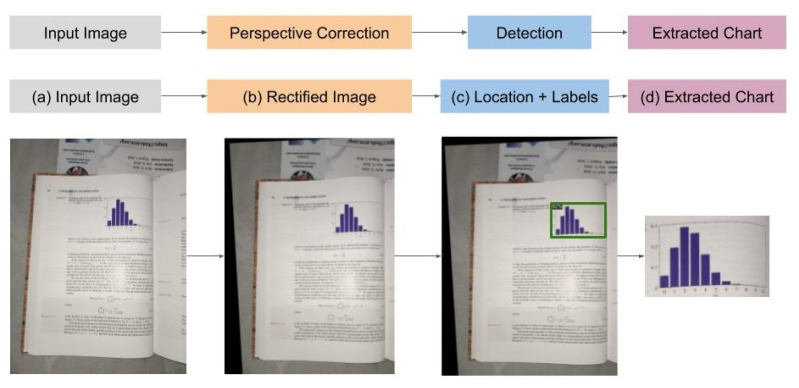
Illustrative example: (**a**) tilted input image, following the (**b**) perspective correction, (**c**) that eased the chart detection (**d**) resulting in a clean-cut bar chart.

The first step in this scenario is the perspective correction of the image, so the image rectification method is used. After rectification of the image, the second step is to use the chart detector to recover the chart position and isolate it. These two steps are shown in Figure 7, with its located bar chart.

In total, these two steps took detection+correction=0.25+0.62=0.87 sec to compute, less time than some camera apps take to save a photography on mobile devices. It is slow to real time frame by frame computation. However, expanding this example, it is possible to use Augmented Reality techniques to superimpose these annotations on the input image directly from the camera stream. Saving the position and using key points of the region makes it possible to track the chart location much faster. In the end, with an extraction method, it is possible to extract the data and highlight it on the image.

Adjustments can be made on the detection model training to recognize charts more accurately, such as introducing different noise options on training, and more training time. However, the results of this use case show that, even with some modifications of state-of-the-art trained models, it is possible to achieve real-time usage of these models. Some hints can be given to the users to position the camera to help the detector. The detection worked without any correction in the simpler cases, but it failed to detect the most tilted charts, and when the detector used the perspective corrected image, it showed a jump in the accuracy of results. In the cases where it is easier to detect, the time of correction could be sparred, but in other hand there is a solution more robust to noise. Our intent with this work is not to show how well the models are trained, but that it is possible to use them on a real-world application of chart recognition chaining these methods.

## 7. Final Remarks and Future Works

The analysis process of a data chart usually has two main steps: to classify the image into a chart type and to extract data from it. These steps already present several solutions, despite the constant need for better approaches to these tasks. Nevertheless, the majority of these solutions only focus on the classification step, and we have noticed that there is a lack of works in the literature linking real-world photos with the task of labeling charts since before labeling. There are many issues to solve, such as locating charts in images and removing camera distortions. This work presented a modern approach in the process of chart recognition, covering classification, detection, and perspective correction, presenting training methods, dataset collection, and methods already in use by the industry for image rectification. It is the first of this kind, bridging the gap of real-world photography and literature research on the field.

A step little-explored in the literature is detecting the data chart in the image. This step is essential if other elements, such as text or pictures, are present in the image that contains the chart, which is quite common in books, newspapers, and magazines. Along with detection, image rectification could be applied to correct the perspective of documents that contain charts. The experiments presented that, for some scenarios, chart recognition already has the technical toolbox available, but it was not organized on an established process. This work hopes to cover this gap, showing that classification, detection, and perspective correction are ready to be used for initial steps of chart recognition, searching for accuracy or time.

The results of the experiments showed that they individually are pairwise with state of the art chart recognition methods, which is important to validate the main contribution of our work. The perspective correction improved by a significant margin (19 detections of 64 without corrective perspective and 31 of 64 using it) the problem of chart detection for a real-world application. Implying that document noise removal approaches can aid the process of chart recognition.

Future works include adding more visualization types for classification, data extraction algorithms on the process, alongside more image corrections. Lightning and noise are aspects unexplored on this work but they have a wide array of solutions on the document analysis field. The evaluation of more perspective correction methods and how to use them have also be considered. A real-world annotated dataset could help with the assessment of more sophisticated methods, as we proposed in the final sections but lacked the data to make it more robust.

The next generation of mobile devices, paired with high bandwidth of 5G, can launch chart recognition in the real world. This novel process of chart recognition covers the literature and expands it to fill some gaps in real-world applications. For instance, it is possible to create augmented reality applications with a process for chart recognition to be used on new scenarios, creating new research opportunities and challenges.

## Figures and Tables

**Table 1:** Dataset summary, with train and test split by each chart type. This dataset is used throughout all steps, with modifications pertinent to each one of them.

Chart Types	Instances		
	Train	Test	Train + Test
Arc	129	26	155
Area	494	87	581
Bar	3883	761	4644
Force Directed Graph	1137	228	1365
Line	2618	529	3147
Parallel Coordinates	702	168	870
Pie	2415	481	2896
Reorderable Matrix	242	42	284
Scatterplot	1797	228	2025
Scatterplot Matrix	837	158	995
Sunburst	540	65	605
Treemap	626	73	699
Wordcloud	2557	276	2833
Total	17,977	3122	21,099

**Table 2:** Results of Chart Classification. Highlight to Xception network with best accuracy results. Blue  cells indicate the right predictions and orange ones indicate high error rate.

Architecture	Learning Rate	Decay	Accuracy–13 Classes	Accuracy–4 classes
**Xception**	**$10^{-4}$**	$10^{-6}$	0.954	0.95
		$10^{-7}$	0.953	0.95
ResNet152	$10^{-5}$	$10^{-6}$	0.948	0.95
	$10^{-4}$	$10^{-7}$	0.947	0.946
VGG19	$10^{-5}$	$10^{-7}$	0.945	0.953
		$10^{-6}$	0.944	0.945
MobileNet	$10^{-4}$	$10^{-6}$	0.926	0.94
	$10^{-5}$	$10^{-7}$	0.922	0.923

**Table 3:** Results of Chart Classification Confusion Matrix of the best model.

	Arc	Area	Bar	Force Directed Graph	Line	Parallel Coordinates	Pie	Reorderable Matrix	Scatterplot	Scatterplot Matrix	Sunburst	Treemap	Wordcloud
Arc	26	0	0	0	0	0	0	0	0	0	0	0	0
Area	0	87	0	0	0	0	0	0	0	0	0	0	0
Bar	0	0	728	0	28	1	0	0	1	2	0	1	0
Force Directed Graph	0	0	0	222	1	1	1	0	0	0	0	1	2
Line	0	2	9	1	511	0	4	0	1	1	0	0	0
Parallel Coordinates	0	0	1	0	0	151	0	0	0	6	0	0	0
Pie	0	0	1	0	1	1	164	0	0	1	0	0	0
Reorderable Matrix	0	1	2	0	0	0	0	477	0	1	0	0	0
Scatterplot	0	0	0	0	0	1	1	0	40	0	0	0	0
Scatterplot Matrix	0	0	2	10	16	10	2	0	1	184	0	0	3
Sunburst	0	0	0	2	0	0	0	10	0	1	50	0	2
Treemap	0	0	3	1	0	0	0	0	1	0	0	66	2
Wordcloud	0	0	1	2	0	0	0	1	0	1	0	0	271

**Table 4:** Results of AP, $AP^{IoU=.5}$, and $AP^{IoU=.75}$ inference values and time. RetinaNet has the best results for any AP value and inference time.

Method	AP	${\mathrm{AP}}^{\mathrm{IoU}=0.5}$	${\mathrm{AP}}^{\mathrm{IoU}=0.75}$	Inference Evaluation Time (s/img)
RetinaNet	81.987	91.127	89.428	0.199285
Faster R-CNN	69.68	79.101	77.428	0.210505

**Table 5:** Average Precision (AP) values for each class in RetinaNet and Faster R-CNN. RetinaNet has the best class AP for all class besides arc and wordcloud.

Class	RetinaNet	Faster R-CNN
Arc	86.513	88.52
Area	78.004	76.447
Bar	87.428	82.334
Force Directed Graph	79.746	45.519
Line	83.494	61.618
Scatterplot Matrix	81.072	70.266
Parallel Coordinates	81.669	61.582
Pie	88.26	83.063
Reorderable Matrix	67.69	61.392
Scatterplot	76.751	66.804
Sunburst	76.84	52.785
Treemap	89.843	73.419
Wordcloud	88.52	88.633

**Table 6:** Results for chart recognition applied to the images of the book based on two approaches: normal and rectified, for full and partial detection. Each image has 15 other versions, varying by slight rotations. Charts (a) and (d) got no detection in any mode. Rectified images got better detection results for other cases.

Mode	Image	Full	Partial
Camera	Chart (a)	–	–
	Chart (b)	9/16	–
	Chart (c)	6/16	4/16
	Chart (d)	–	–
	Chart (e)	–	–
Rectified	Chart (a)	–	–
	Chart (b)	12/16	–
	Chart (c)	12/16	1/16
	Chart (d)	–	–
	Chart (e)	–	6/16
